# Training Load Oscillation and Epigenetic Plasticity: Molecular Pathways Connecting Energy Metabolism and Athletic Personality

**DOI:** 10.3390/ijms27020792

**Published:** 2026-01-13

**Authors:** Dan Cristian Mănescu

**Affiliations:** Department of Physical Education and Sports, Bucharest University of Economic Studies, 010374 Bucharest, Romania; dan.manescu@defs.ase.ro

**Keywords:** training load oscillation, epigenetic plasticity, AMPK, SIRT1, PGC-1α, mTOR, CaMKII, BDNF, DNA methylation, microRNA

## Abstract

Training adaptation involves muscular–metabolic remodeling and personality-linked traits such as motivation, self-regulation, and resilience. This narrative review examines how training load oscillation (TLO)—the deliberate variation in exercise intensity, volume, and substrate availability—may function as a systemic epigenetic stimulus capable of shaping both physiological and psychological adaptation. Fluctuating energetic states reconfigure key energy-sensing pathways (AMPK, mTOR, CaMKII, and SIRT1), thereby potentially influencing DNA methylation, histone acetylation, and microRNA programs linked to PGC-1α and BDNF. This review synthesizes converging evidence suggesting links between these molecular responses and behavioral consistency, cognitive control, and stress tolerance. Building on this literature, a systems model of molecular–behavioral coupling is proposed, in which TLO is hypothesized to entrain phase-shifted AMPK/SIRT1 and mTOR windows, alongside CaMKII intensity pulses and a delayed BDNF crest. The model generates testable predictions—such as amplitude-dependent PGC-1α demethylation, BDNF promoter acetylation, and NR3C1 recalibration under recovery-weighted cycles—and highlights practical implications for timing nutritional, cognitive, and recovery inputs to molecular windows. Understanding TLO as an entrainment signal may help integrate physiology and psychology within a coherent, durable performance strategy. This framework is conceptual in scope and intended to generate testable hypotheses rather than assert definitive mechanisms, providing a structured basis for future empirical investigations integrating molecular, physiological, and behavioral outcomes.

## 1. Introduction

Performance adaptation in athletes extends beyond muscular remodeling or cardiorespiratory efficiency; it also involves behavioral and psychological dimensions such as motivation, self-regulation, and stress resilience. These personality-linked traits determine not only training adherence and consistency but also the ability to translate physiological potential into competitive performance. Traditional approaches in exercise physiology have largely focused on mechanical and metabolic stressors, often treating the psychological component as a separate, downstream effect. However, emerging evidence suggests that these domains are tightly interwoven through shared molecular and signaling pathways [1,2].

During the last decade, the field of sports genomics has advanced our understanding of how genetic predispositions influence trainability, yet purely genetic models explain only a limited fraction of inter-individual variability in performance. The growing recognition of epigenetic mechanisms—including DNA methylation, histone modifications, and microRNA regulation—has shifted attention toward more dynamic and environmentally sensitive molecular processes. These mechanisms respond rapidly to exercise, nutrition, sleep, and psychological stress, thereby creating an interface through which lifestyle modulates gene expression and phenotype. The concept of epigenetic plasticity has thus become central to understanding how transient metabolic and behavioral stimuli may induce long-lasting adaptations relevant to both physiology and personality [3,4,5].

Among the many factors that can trigger epigenetic remodeling, training load oscillation (TLO) represents a particularly powerful and ecologically valid model. TLO refers to the intentional fluctuation of exercise volume, intensity, and energetic substrate availability across micro- and mesocycles. Such oscillatory patterns promote adaptive instability—periods of energetic deficit alternating with recovery and overcompensation—that stimulate signaling cascades such as AMPK–mTOR–CaMKII, SIRT1–PGC-1α, and CREB–BDNF [6,7,8,9]. These pathways regulate not only mitochondrial biogenesis and protein synthesis but also neurotrophic and dopaminergic processes that shape cognitive flexibility, emotional control, and reward sensitivity. Consequently, metabolic variability acts as both a physical and neurobehavioral training stimulus [10,11,12].

The convergence between molecular energetics and behavioral adaptation suggests that athletic personality may be biologically plastic rather than a fixed psychological profile. Epigenetic modifications in genes related to energy metabolism (*PGC-1α*, *NR4A1*), neurotrophic signaling (*BDNF*), and neurotransmission (*COMT*, *DRD2*, *SLC6A4*) have been reported following structured training and environmental challenges. These findings are consistent with the view that consistent exposure to oscillatory training stimuli may recalibrate the molecular architecture underlying motivation and resilience [13,14,15].

Taken together, these observations suggest the need for an integrative framework capable of linking metabolic variability, molecular regulation, and behavioral adaptation. Within this integrative framework, the following Conceptual Assumptions (CA1–CA3) guided the analytical direction of the present review:

**CA1.**  Variability in training load is not random fluctuation but a structured biological input capable of modulating cellular energetic and molecular signaling.

**CA2.**  Epigenetic mechanisms—including DNA methylation, histone modification, and microRNA regulation—constitute the primary mediators translating these metabolic signals into neurobehavioral adaptations.

**CA3.**  The coordination between metabolic and behavioral oscillations can be conceptualized as a unified regulatory process—an *epigenetic resonance*—that sustains both physiological performance and psychological stability.

It is important to note that the present framework is conceptual in nature and does not claim to establish new mechanisms; rather, it organizes converging evidence into a heuristic structure that may guide future hypothesis-driven studies.

Building upon these assumptions, the present review integrates current evidence on the molecular and epigenetic consequences of training load oscillation and advances a systems model of molecular–behavioral coupling. By linking energy metabolism to personality-linked traits of motivation, resilience, and self-regulation, the paper proposes a unifying framework that connects cellular energetics with psychological adaptation, providing a theoretical basis for future precision training and mental conditioning strategies.

## 2. The Concept of Training Load Oscillation

Training Load Oscillation (TLO) can be defined as a structured modulation of exercise intensity, volume, and energetic substrate availability over time, designed to induce successive cycles of metabolic stress and recovery. The concept builds upon the principles of *periodization* and *adaptive perturbation*, where transient disequilibria in homeostasis elicit progressive physiological and psychological adaptations. Unlike linear training models that emphasize cumulative workload, TLO exploits controlled variability as a stimulus for resilience, metabolic flexibility, and neurobehavioral recalibration. This approach aligns with the broader notion of *allostatic regulation*, in which health and performance emerge not from stability, but from the ability to anticipate and recover from perturbation [11,12].

From a physiological standpoint, oscillations in training load modify intracellular energetic states—specifically, the ratios of AMP/ATP, NAD+/NADH, and acetyl-CoA/CoA—which act as sensors for metabolic stress. These oscillations are associated with activation of key signaling pathways such as AMP-activated protein kinase (AMPK), mammalian target of rapamycin (mTOR), calcium/calmodulin-dependent kinase (CaMKII), and sirtuin 1 (SIRT1). Each pathway exerts a distinct yet interdependent role: AMPK promotes catabolic adaptation and mitochondrial biogenesis, mTOR supports anabolic recovery, while SIRT1 and CaMKII integrate redox and calcium signals with epigenetic modifiers. The interplay among these sensors transforms energy fluctuations into molecular information capable of reprogramming gene expression [13].

At the transcriptional level, these dynamic states influence chromatin accessibility through covalent modifications of DNA and histone proteins. Acute energetic stress favors *DNA demethylation* and *histone acetylation* at promoters of oxidative and angiogenic genes such as *PGC-1α*, *VEGF*, and *NR4A1*. Conversely, recovery phases promote chromatin condensation and the restoration of epigenetic balance. The alternation between these states resembles a form of molecular oscillation, where repetitive exposure to variable loads maintains the genome in a responsive, plastic configuration. Such cycles may generate a “metabolic memory” that enhances the organism’s readiness for future challenges [14].

Beyond muscle tissue, TLO also affects the central nervous system and neuroendocrine axes. Exercise-induced metabolic perturbations increase peripheral and cerebral levels of brain-derived neurotrophic factor (BDNF), dopamine, and serotonin, all of which influence mood, motivation, and cognitive control [15]. The amplitude and frequency of training oscillations determine the magnitude of these neurotrophic responses, suggesting that variability itself may act as a neuromodulatory code. Repeated exposure to alternating stress and recovery thus recalibrates the hypothalamic–pituitary–adrenal (HPA) axis, leading to improved emotional stability and resilience under competitive conditions [16].

At the systems level, the TLO paradigm embodies a bi-directional integration of metabolic and behavioral regulation. Metabolic sensors (AMPK, SIRT1) translate energetic variability into transcriptional programs that influence not only mitochondrial and vascular remodeling but also neural plasticity. In parallel, psychological states—motivation, perceived exertion, fatigue—feed back into metabolic regulation via catecholaminergic and serotonergic signaling [17]. This reciprocal coupling implies that training variability can be conceptualized as a cross-level stimulus, simultaneously engaging cellular energetics and higher-order personality traits.

From a theoretical perspective, TLO can be viewed as the operational form of what systems biology terms a *chaotic attractor*: a controlled disorder that promotes adaptability. Within such a framework, the athlete’s physiology does not strive for static optimization but for dynamic coherence, achieved through cycles of destabilization and re-stabilization [18]. When considered alongside epigenetic plasticity, this dynamic coherence implies that performance capacity emerges from the continuous rewriting of molecular and behavioral scripts in response to fluctuating energetic demands [19,20].

In summary, Training Load Oscillation can be conceptualized as a biological principle through which structured variability sustains adaptability.

### 2.1. Scope and Literature Approach

The present article is a narrative review with a conceptual–systems orientation. To ensure transparency while preserving the interpretative nature of the synthesis, a concise description of the literature search strategy, databases consulted, and inclusion criteria is provided in Appendix A.

### 2.2. Operational Parameters of Training Load Oscillation

Although Training Load Oscillation (TLO) is introduced as a structured source of metabolic variability, its empirical use requires a precise operationalization. To support reproducibility and enable cross-study comparisons, TLO can be quantified through three complementary parameters—frequency, amplitude, and duty-cycle—each capturing a distinct dimension of load variability. These parameters can be computed using any consistent metric of training load (e.g., session-RPE × duration, TRIMP, or power-based metrics), and together they define the oscillatory profile capable of driving molecular and behavioral adaptation.

Frequency (F_TLO_)—Frequency denotes the number of biologically distinct *anchor pulses* within a standard 7-day microcycle. Anchor pulses represent sessions that deliver a discrete metabolic or neuromolecular perturbation (e.g., low-glycogen high-intensity work engaging AMPK/SIRT1, fed hypertrophy eliciting mTOR activation, or novelty stimuli activating CaMKII/CREB). Operationally, anchor pulses can be defined as days in which daily load exceeds a data-driven threshold:

$$F_{\mathrm{TLO}}=\#\{d\in [1,7]|L_{d}\geq \bar{L}+k\;\cdot 1\;\mathrm{SD}\left(L\right)\}$$

where $L_{d}$ represents the daily training load (derived from session-RPE × duration, TRIMP, or power-based metrics), $\bar{L}$ is the weekly mean, and SD(L) is the standard deviation. The coefficient k, typically between 0.8 and 1.0, defines how far above the weekly mean a day must rise to constitute a meaningful biological pulse. The operator #{⋅} simply counts the number of days that satisfy this condition. In practice, adaptive microcycles commonly display two to three pulses, usually separated by 36–72 h to maintain the temporal distinction among AMPK/SIRT1-dominant, mTOR-dominant, and CaMKII-linked signaling windows.

Amplitude (A_TLO_)—Amplitude captures the vertical contrast of the oscillation—how strongly the week alternates between high and low metabolic stress. It is computed as the normalized half-range of daily load:

$$A_{\mathrm{TLO}}=\frac{max(L_{d})-min(L_{d})}{2\;\bar{L}}$$

where $L_{max}$ and $L_{min}$ denote the highest and lowest daily loads within the microcycle, while $\bar{L}$ is the weekly mean. The numerator captures the absolute range of variation, and normalization by twice the mean produces a unit-free value that facilitates comparison across athletes and contexts. Moderate amplitudes, typically between 0.30 and 0.60, characterize adaptive weeks; short “shock” microcycles may temporarily reach 0.70–0.80, producing stronger fluctuations in AMPK/SIRT1 and mTOR activity.

Duty-cycle (DC_TLO_)—Duty-cycle represents the fraction of weekly training time performed under high physiological strain. It is operationalized as the proportion of total minutes performed at sRPE ≥ 7:

$$DC_{\mathrm{TLO}}=\frac{\sum_{d}\mathrm{Minutes}(sRPE\geq 7)}{\sum_{d}\mathrm{Total}\;\mathrm{Minutes}}$$

where “Minutes with sRPE ≥ 7” indicates the accumulated time spent above a high-strain perceptual threshold, while “Total Weekly Minutes” denotes the entire training duration within the microcycle. This ratio thus reflects the relative exposure to physiologically demanding work. Balanced microcycles often fall between 0.35 and 0.55, supporting epigenetic responsiveness while preventing excessive allostatic load.

Worked examples—These parameters can be readily mapped onto practical microcycle structures. For instance, an endurance-oriented week featuring a low-glycogen HIIT session (AMPK/SIRT1), a fed strength session (mTOR), and a threshold exposure (CaMKII/CREB) typically yields $F_{\mathrm{TLO}}=2–3$, $A_{\mathrm{TLO}}\approx 70–80\%$, and $DC_{\mathrm{TLO}}\approx 0.45–0.55$, depending on load distribution. Conversely, a strength/skill-focused microcycle with two high-intensity strength pulses spaced by ~72 h commonly produces $F_{\mathrm{TLO}}=2$, $A_{\mathrm{TLO}}\approx 70–80\%$, and $DC_{\mathrm{TLO}}\approx 0.40$. These quantitative profiles illustrate how distinct biological pulses and recovery windows combine to generate the oscillatory landscape through which TLO entrains metabolic and epigenetic pathways.

The proposed operationalization of training load oscillation parameters (frequency, amplitude, and duty cycle) is consistent with established approaches to training load quantification, variability analysis, and non-linear periodization models. Similar principles underpin session-RPE–based load metrics, concepts of training monotony and strain, and the application of complex systems theory to physiological adaptation [11,18].

Collectively, these operational parameters transform TLO from a conceptual descriptor into a measurable construct. They provide the quantitative foundation necessary for designing oscillatory microcycles, testing mechanistic predictions, and linking metabolic variability to epigenetic and behavioral adaptation.

## 3. Molecular Signaling Pathways Induced by TLO

The biological impact of Training Load Oscillation (TLO) is thought to arise from its capacity to modulate the molecular signaling networks that couple cellular energetics to gene expression. In this context, energetic variability functions as an upstream regulator of intracellular sensors such as AMPK, mTOR, CaMKII, and SIRT1, which together translate metabolic fluxes into coordinated transcriptional and epigenetic responses. The oscillatory nature of training stimuli ensures that these pathways are alternately activated and repressed, producing a rhythmic pattern of anabolic and catabolic signaling essential for systemic adaptation [21,22].

### 3.1. AMPK: The Energetic Master Sensor

AMP-activated protein kinase (AMPK) detects the cellular energy state through changes in the AMP/ATP and ADP/ATP ratios. Acute phases of high-intensity or low-glycogen exercise increase AMPK phosphorylation, triggering downstream activation of PGC-1α, the master regulator of mitochondrial biogenesis. In addition to its metabolic effects, AMPK phosphorylates histone deacetylases (HDACs), leading to their nuclear export and facilitating histone acetylation at promoters of oxidative genes. Through this mechanism, energetic stress is directly linked to chromatin remodeling and gene transcription. Repeated AMPK activation during oscillatory training cycles may establish a pattern of *epigenetic anticipation*, enabling faster transcriptional responses to future energetic challenges [23,24,25].

### 3.2. mTOR: Anabolic Recovery and Molecular Memory

Conversely, recovery and high-energy availability phases stimulate the mammalian target of rapamycin complex 1 (mTORC1), which promotes protein synthesis, ribosomal biogenesis, and growth-related gene expression. mTORC1 activity is sensitive to amino acid availability, mechanical load, and insulin signaling, representing the anabolic counterpart to AMPK’s catabolic influence. The alternation between AMPK and mTOR activation across TLO cycles forms a dynamic *metabolic switch* that governs the balance between energy conservation and tissue remodeling. This reciprocal regulation may optimize physical adaptation and may contribute to synaptic plasticity, learning processes within the brain, where mTOR signaling is critical for memory consolidation and motivational reinforcement [26,27,28].

### 3.3. CaMKII: Calcium and Neuroplastic Integration

Fluctuations in training intensity modify cytosolic calcium levels, activating calcium/calmodulin-dependent protein kinase II (CaMKII). This kinase regulates both metabolic enzymes and transcription factors, including CREB (cAMP response element-binding protein), which is required for the induction of brain-derived neurotrophic factor (BDNF). BDNF expression enhances neurogenesis, synaptic remodeling, and motivational drive, linking muscular effort to cognitive adaptation. Although CaMKII activation is a shared response to intensity-driven stimuli, its functional roles differ markedly across tissues. In skeletal muscle, CaMKII primarily regulates metabolic enzymes and contributes to excitation–transcription coupling. By contrast, the α and β isoforms dominate in hippocampal and cortical neurons, where they are essential for CREB-dependent induction of BDNF, synaptic remodeling, and learning-related plasticity. Most mechanistic evidence for CaMKII–CREB–BDNF coupling therefore derives from rodent neural tissue, and these central dynamics cannot be assumed to mirror peripheral CaMKII signaling in muscle. Within this review, references to CaMKII-linked neuroplasticity pertain specifically to central CaMKII-α/β mechanisms, whereas intensity-induced CaMKII activity in muscle represents a distinct, peripheral pathway [29,30,31].

### 3.4. SIRT1 and Redox Coupling

Another key mediator of the TLO response is sirtuin 1 (SIRT1), a NAD^+^-dependent deacetylase that integrates redox state and metabolic flux with gene regulation. In conditions of energetic stress, elevated NAD^+^ levels activate SIRT1, which deacetylates PGC-1α, FOXO, and histone substrates, promoting oxidative metabolism and antioxidant defense. SIRT1 also modulates circadian transcriptional programs and interacts with AMPK in a positive feedback loop, reinforcing mitochondrial efficiency and metabolic flexibility. The oscillation between reduced and oxidized NAD^+^ pools during TLO cycles thereby imposes rhythmic epigenetic control on energy- and stress-responsive genes [32,33,34].

### 3.5. Integrative Network Dynamics

Collectively, AMPK, mTOR, CaMKII, and SIRT1 constitute a metabolic signaling quartet that governs the adaptive phenotype. Their oscillatory activation in response to TLO ensures that neither anabolic nor catabolic processes dominate for extended periods, preserving systemic homeodynamics. Beyond the classical muscle-centered view, these signals propagate to multiple organs—liver, adipose tissue, brain—via circulating metabolites, exosomes, and cytokines. Such inter-tissue communication supports the hypothesis that TLO operates as a whole-body training signal, coordinating peripheral energy metabolism with central motivational and cognitive processes [35,36,37].

### 3.6. From Signal Oscillation to Epigenetic Plasticity

The recurring activation–recovery cycles inherent in TLO represent an internal chronobiological rhythm capable of entraining epigenetic enzymes. AMPK and SIRT1 regulate the availability of cofactors such as acetyl-CoA, NAD^+^, and S-adenosylmethionine (SAM), directly influencing the activity of histone acetyltransferases (HATs), deacetylases (HDACs), and DNA methyltransferases (DNMTs). The resulting fluctuations in chromatin structure facilitate transient gene accessibility, enabling coordinated expression of genes related to both physical and behavioral adaptation. This biochemical coupling establishes the foundation for *epigenetic resonance*—a state in which energy metabolism and personality-linked traits coevolve through shared molecular rhythms.

In summary, the molecular architecture activated by TLO transforms energetic variability into structured biological information. Through its influence on AMPK, mTOR, CaMKII, and SIRT1, TLO orchestrates a complex network that synchronizes mitochondrial function, neural plasticity, and motivational regulation.

**Figure 1:**
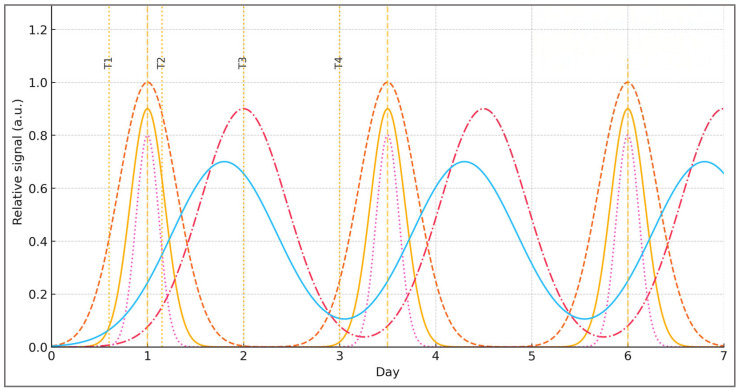
Training Load Oscillation entrains phase-shifted molecular responses (schematic). Illus-trative multi-trace timecourses show TLO session impulses, antiphased AMPK/SIRT1 and mTOR windows, intensity-locked CaMKII spikes, and a delayed BDNF crest aligned with consolidation. Vertical markers illustrate standardized sampling windows (T1–T4) around the first pulse. Curves are schematic and not empirical data.

To make the oscillatory logic explicit, Figure 1 illustrates a conceptual time-course in which training load oscillation (TLO) entrains phase-shifted molecular responses: AMPK/SIRT1 peak under energetic stress while mTOR dominates recovery, with CaMKII intensity pulses and a delayed BDNF crest.

While Figure 1 represents these oscillatory signatures as a unified conceptual waveform, the underlying biology is distributed across tissues. Peripheral CaMKII activity in skeletal muscle primarily supports metabolic and excitation–transcription functions, whereas the delayed neurotrophic crest depicted in the figure reflects central CaMKII-α/β–CREB–BDNF signaling within hippocampal and cortical circuits. This distinction preserves conceptual clarity without implying that peripheral and central CaMKII dynamics are interchangeable.

These oscillatory signaling patterns provide the molecular context within which epigenetic plasticity can emerge.

## 4. Epigenetic Mechanisms Bridging Energy and Personality

The translation of metabolic signaling into long-term adaptation depends on the ability of the epigenome to record and reinterpret transient energetic stimuli. Epigenetic regulation encompasses covalent modifications to DNA and histone proteins, as well as the modulation of gene expression through non-coding RNAs such as microRNAs. These mechanisms provide a reversible interface through which exercise and environmental challenges exert enduring molecular and behavioral effects. Training load oscillation (TLO), by generating rhythmic activation of AMPK, mTOR, CaMKII, and SIRT1, continuously alters the intracellular availability of metabolic cofactors—acetyl-CoA, NAD^+^, and S-adenosylmethionine (SAM)—that determine chromatin accessibility and transcriptional potential. Thus, energy flux can be conceptualized as an epigenetic signal capable of shaping both cellular function and personality-linked behavior [38,39,40].

### 4.1. DNA Methylation Dynamics

DNA methylation, catalyzed by DNA methyltransferases (DNMT1, DNMT3A/B), regulates gene expression by modifying cytosine residues within CpG islands. Exercise induces locus-specific changes in DNA methylation within skeletal muscle, brain, and peripheral blood mononuclear cells. Hypomethylation of the PGC-1α promoter following acute or chronic exercise enhances oxidative capacity, while methylation changes in the BDNF and NR4A1 genes modulate neuroplastic and motivational pathways. Importantly, these alterations exhibit partial reversibility, indicating a dynamic equilibrium rather than a fixed imprint. The magnitude and persistence of methylation shifts are proportional to the amplitude and frequency of energetic oscillations induced by TLO, suggesting a dose–response relationship between training variability and epigenetic remodeling.

In the neural context, differential methylation of genes encoding dopaminergic and serotonergic components (COMT, DRD2, SLC6A4) has been associated with changes in mood regulation, impulsivity, and cognitive performance. These findings suggest that metabolic challenges mediated through TLO can indirectly recalibrate neurochemical systems governing personality traits relevant to athletic success [41,42,43].

### 4.2. Histone Modifications and Chromatin Remodeling

Histone acetylation and methylation dynamically regulate chromatin structure and transcriptional activity. Exercise-induced increases in histone H3 acetylation (H3K9ac, H3K27ac) enhance accessibility of genes involved in mitochondrial biogenesis, angiogenesis, and neurogenesis. AMPK and SIRT1 influence these processes by phosphorylating or deacetylating histone-modifying enzymes, thereby linking energy status to chromatin conformation. During high-intensity or low-glycogen phases of TLO, elevated AMP/ATP and NAD^+^ levels favor deacetylation and activation of oxidative pathways. Recovery phases restore acetyl-CoA availability, supporting HAT activity and anabolic transcription. This alternation creates a cyclical pattern of histone modification that mirrors training oscillations, maintaining the genome in a flexible, responsive state.

Furthermore, histone methylation at lysine residues (H3K4me3, H3K27me3) adjusts the balance between activation and repression of stress- and motivation-related genes [35]. In animal models, exercise-induced changes in these marks within the hippocampus and nucleus accumbens correlate with enhanced learning, emotional regulation, and persistence—traits fundamental to athletic personality [44,45,46].

### 4.3. MicroRNAs as Translators of Energetic Stress

MicroRNAs (miRNAs) constitute an additional epigenetic layer that fine-tunes gene expression post-transcriptionally. Numerous miRNAs respond acutely to exercise and metabolic perturbation, including miR-1, miR-133a/b, miR-206 (myomiRs), and miR-132 and miR-134 (neuro-miRs). These molecules modulate networks governing muscle plasticity, neurogenesis, and neurotransmitter signaling. For example, upregulation of miR-132 promotes BDNF expression and synaptic remodeling, while miR-134 regulates dendritic spine morphology and emotional reactivity. The circulating profile of these miRNAs reflects systemic adaptation and can serve as a biomarker of both physical and psychological responses to TLO.

The rhythmic activation of AMPK–SIRT1 and CREB–BDNF pathways during training oscillation may entrain miRNA expression cycles, establishing a molecular resonance between energy metabolism and cognitive-motivational states. This resonance provides a plausible mechanism for how epigenetic plasticity bridges metabolic efficiency and behavioral consistency [47,48,49,50].

### 4.4. Integrative View: Epigenetic Coupling Between Energy and Behavior

The combined action of DNA methylation, histone modification, and microRNA regulation forms a multilayered system of epigenetic coupling through which fluctuating metabolic inputs may shape both physiological and behavioral adaptation. Under conditions of structured training load oscillation, these mechanisms collectively reinforce transcriptional programs associated with metabolic flexibility, motivation, and resilience [51,52,53].

Representative evidence supporting this integrative view is summarized in Table 1, which maps exercise-induced epigenetic modifications across metabolic and neurobehavioral pathways, highlighting convergent patterns observed in both human and animal studies.

The studies summarized in Table 1 include both human and animal models. Human studies are explicitly indicated, while animal studies are included when providing mechanistic insight not readily accessible in human research.

### 4.5. Quantitative Snapshot of Representative Epigenetic Effects

Although narrative in scope, the present review benefits from anchoring its conceptual claims in the quantitative patterns reported across the most influential exercise–epigenetic studies. Rather than functioning as a meta-analysis, the aim of this subsection is to provide orders of magnitude, unit-specific effect expressions, and temporal alignment (T1–T4) for representative findings in DNA methylation, histone marks, microRNAs, and gene expression. The goal is transparency, not aggregation: tissues, assays, and sampling windows differ substantially across studies, and no statistical pooling is attempted. Still, certain regularities emerge with sufficient clarity to inform the broader systems framework.

Across acute endurance and sprint protocols, DNA methylation typically shifts by 2–10 percentage points at exercise-responsive loci such as *PGC-1α*, *PDK4*, *BDNF*, or *NR4A1*, most consistently in the T2 window (2–4 h post-exercise). These changes generally invert or consolidate by T3 depending on substrate status and recovery structure. During longitudinal training, methylation effects accumulate: reductions of 3–15 p.p. have been reported at oxidative and neurotrophic loci, with some marks (notably in *PGC-1α* or *MEF2A*) retaining partial memory weeks after detraining.

Acute exercise also elicits log_2_ fold-changes of ~0.5–2.0 in myomiRs (*miR-1*, *miR-133a*, *miR-206*) and neuro-miRs (*miR-132*, *miR-134*), again peaking in T2 and showing partial normalization by T3–T4. In animal hippocampus and, to a lesser extent, human peripheral tissues, histone acetylation (H3K9ac, H3K27ac) typically increases by 20–80% (or ~0.3–1.0 log_2_FC), particularly after novelty- or intensity-rich stimuli. Transcriptomic responses follow a similar temporal logic: acute oxidative signatures are usually seen in T2, with transcriptional consolidation in pathways related to mitochondrial function and neuroplasticity unfolding over T3–T4.

To provide an anchor for the conceptual framework, Table 2 synthesizes representative unit-specific magnitudes from key exercise–epigenetic studies, organized by tissue, pathway, direction, and temporal window (T1–T4). Values reflect orders of magnitude re-ported in the literature; heterogeneity in assays, sampling protocols, and tissues precludes statistical pooling. The intent is contextualization rather than aggregation, illustrating the scale and timing of epigenetic responses compatible with oscillatory training.

These values should be interpreted as indicative rather than directly comparable, given heterogeneity in tissues, assays, and sampling protocols. The purpose is not statistical aggregation but contextualization—providing unit-specific scales that clarify how oscillatory training stimuli map onto epigenetic dynamics across T1–T4. The pattern that emerges is consistent with the framework developed in Section 2, Section 3, Section 4, Section 5, Section 6 and Section 7: acute metabolic perturbations typically appear in T2, consolidation-related changes in T3, and stabilization in T4, with multi-week memory effects accumulating under structured variability.

## 5. From Molecular Plasticity to Behavioral Adaptation

The transition from molecular adaptation to behavioral expression represents the final step in the integrative cascade initiated by training load oscillation (TLO). Through repeated cycles of energetic challenge and recovery, molecular networks governing energy metabolism become entrained with neural circuits responsible for motivation, self-regulation, and emotional balance [58]. This process, which may be termed epigenetic behavioral coupling, offers a conceptual explanation for how physical training influences personality traits relevant to performance consistency.

### 5.1. Neurotrophic Integration and Motivation

Among the numerous molecular mediators linking exercise to psychological adaptation, brain-derived neurotrophic factor (BDNF) occupies a central position. Much of the mechanistic understanding of how CaMKII, CREB, and BDNF interact to support motivational plasticity derives from rodent hippocampal and cortical studies, where the CaMKII-α and CaMKII-β isoforms predominate. These central dynamics should not be assumed to parallel peripheral skeletal-muscle CaMKII signaling, which largely governs metabolic and excitation–transcription processes. Accordingly, neural interpretations throughout this section refer specifically to central CaMKII isoforms and their CREB-dependent regulation of BDNF.

BDNF regulates synaptic plasticity, neurogenesis, and dopaminergic signaling in brain regions implicated in motivation and reward, including the prefrontal cortex and nucleus accumbens. Oscillatory training stimuli elevate circulating and central BDNF concentrations through the combined activation of AMPK, CaMKII, and CREB. These changes are consistent with enhanced neural plasticity and may contribute to greater persistence, goal-directed behavior, and resistance to fatigue.

Epigenetic regulation of the *BDNF* gene, particularly through promoter demethylation and increased H3 acetylation, underlies the long-term consolidation of these effects. Experimental studies have shown that exercise-induced BDNF upregulation parallels improvements in learning and stress coping, suggesting that motivation itself may represent an acquired phenotype shaped by molecular memory within neural circuits. Through this pathway, metabolic variability imposed by TLO may contribute to a self-reinforcing cycle of effort and reward sensitivity [59,60,61,62].

### 5.2. Dopaminergic and Serotonergic Modulation of Self-Regulation

The dopaminergic and serotonergic systems are key regulators of behavioral control, reward evaluation, and emotional stability—all crucial elements of athletic personality. Variations in dopamine synthesis, receptor density, and reuptake efficiency have been associated with differences in behavioral control, although most fine-grained epigenetic insights—particularly at COMT (catechol-O-methyltransferase), DRD2 (dopamine D2 receptor), and SLC6A4 (serotonin transporter)—originate from human blood or saliva studies, whereas circuit-level interpretations often rely on animal data [63,64].

TLO magnifies these effects by alternating high- and low-stress phases, thereby engaging both dopaminergic arousal and serotonergic recovery mechanisms. Over time, the brain adapts to this rhythmic stress exposure by optimizing receptor density and neurotransmitter turnover. DNA methylation changes in the DRD2 and SLC6A4 promoters have been observed following exercise and stress conditioning paradigms, correlating with enhanced emotional regulation and reduced anxiety. Such evidence supports the hypothesis that metabolic oscillations can entrain the molecular substrates of self-regulation, transforming transient neurochemical events into durable behavioral traits [65,66].

### 5.3. Cortisol Dynamics and Stress Resilience

A defining component of athletic performance is the capacity to tolerate and recover from stress. The hypothalamic–pituitary–adrenal (HPA) axis plays a central role in this process, and its responsiveness can be reshaped through repetitive exposure to fluctuating energetic and psychological loads. TLO introduces controlled perturbations of cortisol and catecholamine levels, producing adaptive recalibration rather than chronic elevation. In parallel, glucocorticoid receptor signaling interacts with AMPK and SIRT1 pathways, influencing histone deacetylation and transcriptional control of stress-related genes.

Epigenetic modifications within the NR3C1 (glucocorticoid receptor) gene have been linked to improved stress tolerance and reduced allostatic load in trained individuals. Most mechanistic links between glucocorticoid-receptor methylation, HPA-axis recalibration, and behavioral resilience come from peripheral blood or saliva studies, and the correspondence between central and peripheral *NR3C1* regulation remains an open empirical question. These findings suggest that stress resilience in trained individuals may reflect both psychological adaptation and molecular conditioning. The cyclic activation–recovery pattern characteristic of TLO thus mirrors the oscillatory operation of the HPA axis, maintaining homeostasis through rhythmic epigenetic recalibration [67,68,69].

### 5.4. The Behavioral Phenotype of Metabolic Flexibility

When integrated, these neurotrophic, neurotransmitter, and hormonal mechanisms are associated with a behavioral phenotype characterized by persistence, adaptive focus, and emotional stability. This metabolically flexible personality enables athletes to modulate effort, attention, and recovery according to context, optimizing both physiological and psychological efficiency. From a systems perspective, this phenotype emerges as a higher-order manifestation of epigenetic plasticity—a process through which the organism learns to anticipate and exploit variability rather than resist it [70,71].

Behavioral studies indicate that individuals exposed to oscillatory or variable training paradigms exhibit superior adherence, intrinsic motivation, and resilience under competitive stress compared with those following monotonous regimens. The observed differences in coping strategies and motivational orientation can thus be viewed as the behavioral output of the molecular–epigenetic mechanisms previously described [72,73].

### 5.5. Conceptual Integration

The alignment between metabolic and behavioral plasticity embodies the principle of epigenetic resonance, in which oscillatory energetic inputs synchronize gene networks across tissues and systems. In this framework, motivation, discipline, and resilience are not abstract traits but quantifiable outcomes of molecular coherence between peripheral metabolism and central neurobiology. This reconceptualization bridges physiology and psychology, suggesting that the most sustainable form of performance emerges when energetic and cognitive rhythms are harmonized through structured variability.

Given the integrative and cross-level nature of the evidence reviewed, the conceptual framework developed in the next section should be interpreted as heuristic rather than mechanistic. Its aim is to organize converging observations and generate empirically testable hypotheses, not to assert definitive causal pathways.

## 6. A Systems Model of Molecular–Behavioral Coupling

The preceding sections outline a continuum from metabolic perturbation to behavioral adaptation, mediated through layers of molecular and epigenetic regulation. To capture these interactions in an integrative framework, a systems model of molecular–behavioral coupling is proposed, in which energetic variability acts as a universal input and behavioral coherence emerges as an output. The model positions the epigenome as the central translator, converting biochemical fluctuations into coordinated physiological and psychological states.

### 6.1. Structural Overview of the Model

**Figure 2:**
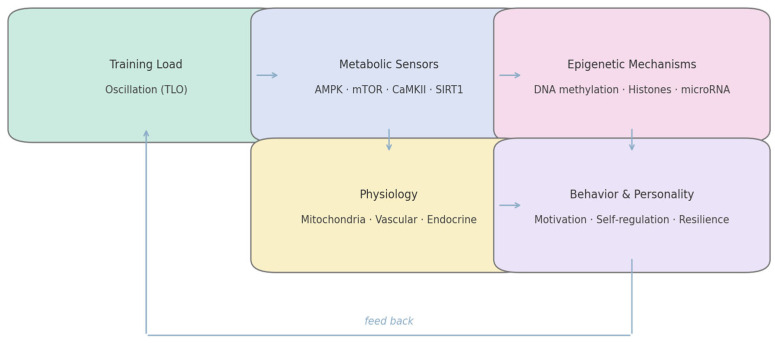
Closed-loop model linking energy sensing, epigenetics, physiology, and behavior. The loop formalizes how motivation/self-regulation modulate realized training load, closing the molecular–behavioral circuit. This figure is schematic and intended for conceptual illustration rather than empirical quantification.

Training load oscillation (TLO) serves as the initiating input of the model. Each oscillatory cycle—alternating metabolic depletion and replenishment—modulates cellular energy sensors (AMPK, mTOR, CaMKII, SIRT1). These pathways, in turn, regulate chromatin-modifying enzymes, producing transient shifts in DNA methylation, histone acetylation, and microRNA expression. The resulting epigenetic landscape orchestrates gene programs for mitochondrial function, stress signaling, and neuroplasticity, which shape motivation, affect regulation, and executive control; these behavioral tendencies then feed back to the realized training load via adherence and effort regulation. The closed-loop architecture is summarized in Figure 2.

By placing behavior in the same lane as epigenetics and physiology, the model treats motivation as a state variable with molecular inputs. This suggests cross-level interventions (e.g., nutritional NAD^+^ support + attentional training) can be synergistic if timed to the same oscillatory phase—i.e., a practical handle on “resonance” rather than a metaphor.

This dynamic can be conceptualized as a closed-loop system: metabolic variability generates epigenetic modulation; epigenetic modulation alters gene expression; gene expression redefines behavioral tendencies; and behavioral tendencies feedback to influence metabolic regulation through effort, adherence, and cognitive-emotional engagement. Thus, the athlete is both architect and product of their molecular environment—a self-regulating organism whose personality traits mirror the stability of their bioenergetic rhythms [74,75].

### 6.2. Functional Domains and Cross-Level Integration

The model integrates three interdependent domains. The first is the metabolic domain, encompassing the interplay between energy availability, nutrient flux, and redox balance. The second is the epigenetic domain, where metabolic cofactors such as acetyl-CoA, NAD^+^, and SAM modulate the activity of chromatin enzymes. The third is the behavioral domain, in which neural circuits translate molecular signals into patterns of motivation, resilience, and self-regulation. The continuous exchange of information among these domains creates a form of biological coherence—an alignment of energetic, molecular, and psychological rhythms that supports optimal adaptation.

In this view, health and performance arise not from maximizing any single domain but from maintaining resonant synchronization among them. Perturbations that disrupt one level (e.g., chronic caloric restriction, monotony, or psychological burnout) desynchronize the system, leading to maladaptive responses such as overtraining or motivational fatigue. Conversely, structured oscillations within an optimal range sustain adaptability by continuously updating the molecular–behavioral interface [51,76,77,78].

### 6.3. Epigenetic Resonance: The Core Mechanism

Epigenetic resonance is used here to refer to the entrainment of gene-regulatory dynamics to rhythmic energetic inputs. Repeated cycles of metabolic stress and recovery generate predictable oscillations in chromatin accessibility and transcriptional responsiveness. When these oscillations are aligned with behavioral and circadian rhythms, they produce a coherent adaptive state characterized by efficient energy use, emotional stability, and consistent performance. In contrast, irregular or excessive oscillations can create discordant epigenetic patterns, impairing adaptation.

This entrainment mechanism may provide a unifying principle that explains why variability—when appropriately structured—is more beneficial than constancy. It allows the organism to encode temporal patterns of experience into molecular memory, facilitating learning across both physiological and behavioral dimensions [79,80].

To operationalize this concept, a prototype Resonance Index (RI) is introduced that captures the balance between epigenetic signals associated with adaptive plasticity and those reflecting stress-driven maladaptation. In conceptual terms, adaptive responses include reduced methylation at *PPARGC1A* and *BDNF*, increased SIRT1 activity, enhanced H3K27 acetylation, and elevated miR-132, whereas maladaptive tendencies include increased methylation at *NR3C1* or *SLC6A4*. Although not intended as a prescriptive diagnostic tool, the RI provides a useful composite expression of these tendencies:

$$RI=z\left(↓{DNAm}_{PPARGC1A}\right)+z\left(↓{DNAm}_{BDNF}\right)+z\left(↑SIRT1\right)+z\left(↑H3K27ac\right)\;+z(↑miR132)-\left[z(↑{DNAm}_{NR3C1})+z(↑{DNAm}_{SLC6A4})\right]$$

Each term represents a standardized deviation (z-score) in the direction biologically interpreted as favorable or unfavorable. A rising RI across successive microcycles would indicate increasing alignment between energetic inputs and epigenetic responsiveness, whereas a declining RI—despite similar external workloads—would signal loss of coherence within the molecular–behavioral system. In this way, epigenetic resonance becomes a falsifiable construct that can be examined using repeated sampling across T1–T4 windows.

**Figure 3:**
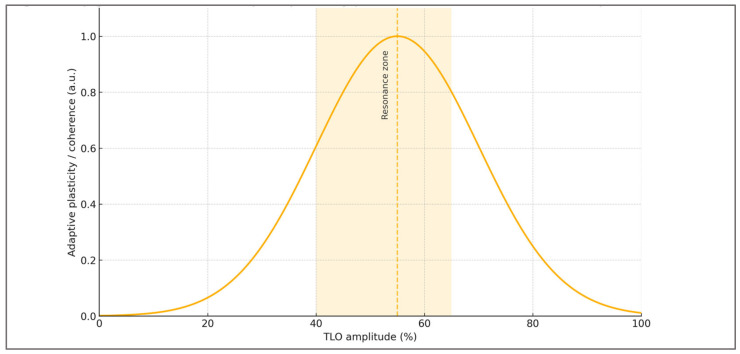
Epigenetic resonance: adaptive plasticity peaks at intermediate oscillation amplitude (schematic). Conceptual response curve relating TLO amplitude to a composite of metabolic–epigenetic–behavioral coherence. Under- and over-oscillation reduce plasticity; an intermediate “optimal adaptive zone” maximizes responsiveness. The shaded area indicates the proposed optimal oscillation (“resonance”) zone. This figure is schematic and intended for conceptual illustration rather than empirical quantification.

Building on the closed-loop model, Figure 3 depicts the hypothesized non-linear response to oscillation amplitude—an optimal adaptive zone where genomic, epigenetic, and behavioral adaptability peak. Both under- and over-oscillation reduce plasticity, irrespective of similar average workloads.

Viewed through this lens, oscillatory training becomes a matter of phase-engineering rather than peak accumulation. What ultimately governs plasticity is not the magnitude of the hardest sessions, but the coherence of the sequence linking stress, recovery, novelty, and consolidation. This perspective naturally motivates individualized calibration: the same absolute load can occupy different positions on the adaptive response curve depending on the athlete’s redox background, sleep alignment, cognitive load, and overall adaptive tone. In this sense, amplitude is not a fixed characteristic of a program but an emergent property of the organism–environment interaction.

### 6.4. Implications for Measurement and Intervention

The systems model implies that markers of adaptation should be evaluated across multiple levels simultaneously. Metabolic indicators (lactate kinetics, respiratory exchange ratio, substrate utilization) should be complemented by molecular biomarkers (DNA methylation at PGC-1α, BDNF, COMT loci; circulating miR-132 and miR-206) and psychological metrics (motivation, resilience, affect regulation). Correlating these layers could enable the identification of individual resonance profiles—unique signatures of bioenergetic and behavioral synchronization.

Such profiling may serve as the foundation for precision training interventions that adapt load variability, nutritional timing, and psychological conditioning to maintain epigenetic coherence. In practical terms, this approach moves beyond the dichotomy of “physical” and “mental” training, treating both as manifestations of a single regulatory continuum.

### 6.5. Theoretical and Practical Significance

By situating personality within a systems physiology framework, the molecular–behavioral coupling model challenges conventional distinctions between biology and psychology. It suggests that personality traits can evolve through iterative molecular learning processes—an idea consistent with emerging data in neuroepigenetics and psychogenomics. In athletes, this model provides a rationale for why disciplined yet flexible training regimens foster both resilience and performance longevity [81,82,83].

Practically, the model supports the design of “adaptive periodization” strategies that intentionally vary metabolic stress to optimize both physical and psychological outcomes. It also offers explanatory power for phenomena such as the “runner’s high” or “flow state,” which may reflect temporary states of maximal epigenetic resonance between energy metabolism and cognitive control networks [84,85,86,87].

Interpretive scope and limitations of the model: while the proposed systems model integrates converging evidence across molecular, physiological, and behavioral domains, it is important to emphasize that several of the inferred links—particularly those connecting epigenetic modulation to complex exercise-related personality traits such as motivation, self-regulation, and resilience—remain inferential rather than causally established. Current human evidence in this area is predominantly associative, and the directionality of the proposed Training Load Oscillation (TLO) → epigenetic regulation → behavioral adaptation pathway cannot be definitively resolved based on existing data. Moreover, a substantial portion of the mechanistic evidence informing the model derives from animal studies, especially with respect to hippocampal CaMKII–BDNF signaling, which provides valuable insight into neuroplastic processes but cannot be directly extrapolated to peripheral tissues such as skeletal muscle or to integrated brain function during human exercise. Accordingly, the present framework should be interpreted as hypothesis-generating rather than mechanistic, underscoring the need for tissue-specific, longitudinal, and controlled experimental studies in human participants to validate the proposed molecular–behavioral coupling and its temporal dynamics.

In summary, the systems model of molecular–behavioral coupling conceptualizes human performance as a product of synchronized variability. By integrating metabolic, epigenetic, and behavioral rhythms, it provides a coherent theoretical scaffold for understanding how training load oscillation shapes both the body and the mind.

Building upon this conceptual foundation, the model can also inform empirical and applied perspectives. It delineates measurable relationships between metabolic oscillations and behavioral adaptation, which may guide methodological innovation aimed at linking molecular mechanisms with observable training outcomes. Based on this integrative logic, several conceptual propositions can be formulated to describe how variability in training load may translate into adaptive molecular and behavioral responses. These propositions are summarized in Table 3, which specifies the expected mechanistic relationships and their relevance for future research and applied practice.

Following the conceptual propositions summarized in the table above, the next logical step is to identify empirical directions through which the molecular–behavioral coupling model can be examined in practice. To this end, Table 4 outlines methodological approaches and experimental contexts suitable for exploring the mechanisms of epigenetic resonance and its role in adaptive responses to training load oscillation.

## 7. Implications for Precision and Mental Conditioning

The systems view of molecular–behavioral coupling outlined above offers a conceptual and practical foundation for a new generation of precision training models. These models recognize that the optimal adaptation of an athlete is not defined solely by the magnitude of physical stimuli but by the synchronization between energetic, molecular, and psychological rhythms. The aim of training; therefore, is not to impose greater stress but to achieve coherence—a state in which metabolic, neural, and behavioral oscillations operate in resonance.

This section integrates evidence-supported principles from training physiology, neuroplasticity, and behavioral science with emerging, hypothesis-generating propositions derived from the proposed systems framework. Accordingly, applied recommendations grounded in existing empirical data are distinguished from conceptual extrapolations intended to guide future experimental testing rather than to serve as prescriptive protocols.

### 7.1. Precision Training as Molecular Synchronization

In this framework, precision training does not mean adding more work; it means shaping the oscillation so that its amplitude, frequency, and duty-cycle (stress versus recovery) are phase-aligned with the organism’s molecular windows of plasticity. External load acts as an entrainment signal for the AMPK–mTOR–CaMKII–SIRT1 quartet and the epigenetic machinery they control. When the waveform is clear, metabolism, chromatin, and behavior cohere; when it is muddled, the same workload turns into noise [88,89,90,91].

At the microcycle level, think of the week as a waveform with two or three anchor pulses that are biologically distinct and separated by space that protects their effects. A practical pattern is to open the week with a high-intensity metabolic stimulus—optionally under low-glycogen—to engage AMPK/SIRT1 and CaMKII; follow with a low-volume skill or easy-aerobic day that preserves signaling memory; place a fed strength/hypertrophy session mid-week to deliver a clean mTOR pulse; restore transcriptional consolidation with a recovery or mobility day; and close with a threshold or long-aerobic exposure that reinforces oxidative programs, leaving technique and recovery to maintain rhythm without adding noise. Across mesocycles, novelty is introduced deliberately to refresh CaMKII/CREB–BDNF signaling, amplitude is scaled gradually, and deloads are inserted before responsiveness fades [57,92,93,94,95,96].

Adjustment follows simple real-time rules. If perceived effort rises while output falls at a stable external load, reduce amplitude or expand the recovery fraction. If the week shows no clear highs and lows, increase contrast—make the hard work truly hard and the easy work genuinely easy—or add a single novelty pulse. If there are signs of catabolic carry-over (sleep disruption, appetite loss, elevated baseline tension), remove one pulse and re-anchor circadian habits before touching intensity. Above all, keep each day’s intent singular so that pulses do not collide biologically [97].

This load architecture must be timed with mental work and substrate state. Cognitive blocks prime or quiet the system at specific phases, while nutrition and recovery set the cofactor landscape (NAD^+^, acetyl-CoA, SAM, sleep) so each pulse carries its intended molecular meaning. Precision, in this sense, is the accumulation of coherence rather than of fatigue: clear AMPK/SIRT1 and CaMKII windows followed by protected mTOR and learning windows yield not only better tissues but a stable behavioral phenotype capable of reproducing performance under pressure [98,99].

### 7.2. Mental Conditioning as Behavioral Resonance

Mental conditioning is not an optional add-on to physical preparation; it is the behavioral oscillator that must be phase-aligned with training load oscillation and the molecular windows it opens. The target is the prefrontal–limbic–striatal circuitry that governs attention, valuation, and effort. Through CREB/BDNF-dependent plasticity and the modulation of dopaminergic (COMT/DRD2), serotonergic (SLC6A4), and HPA (NR3C1) pathways, well-timed mental practice can make cognitive effort amplify the very epigenetic processes that sustain physiological adaptation [100,101,102,103].

In practice, a small toolkit covers most needs. Attentional control—learning to switch deliberately between narrow task focus and open monitoring—reduces interoceptive noise and stabilizes pacing and perceived exertion. Mindfulness/acceptance trains non-reactivity at high arousal, down-tunes stress reactivity, and supports BDNF-linked flexibility. Imagery and motor simulation strengthen task templates and are most effective when placed into early consolidation windows. Strategic self-talk and implementation intentions turn abstract goals into cue-bound actions, lowering executive load under catecholamine pressure. HRV-biofeedback and breathwork raise vagal tone and align autonomic state with the intended task difficulty [104,105].

Timing is the difference between an exercise and an effect. A short pre-session priming (6–10 min of focus plus brief imagery) sharpens control before the catecholamine surge. During demanding segments, pre-rehearsed cue switches prevent tunnel vision without diluting drive. Immediately post-session, a short down-shift (mindfulness or HRV coherence) facilitates BDNF-mediated consolidation while AMPK/SIRT1 windows are still open. On the next morning, a brief retrieval or imagery rehearsal stabilizes the updated pacing or technique. On recovery days, light cognitive maintenance—open monitoring and concise journaling of cues and RPE—preserves coherence without adding load. As with physical training, mental stress is periodized: frequent micro-doses and occasional heavier blocks only when recovery and nutrition support them [106].

Monitoring should mirror this logic. Practical pairings align state motivation/affect with BDNF (DNAm/protein) and sleep/HRV with NR3C1 methylation across T1–T4 sampling windows. Phase-consistent improvements indicate successful entrainment; flattening or inversion suggests impending maladaptation and calls for retiming or reducing cognitive load. When dose and timing respect the metabolic windows, mental work becomes a low-cost amplifier of both performance capacity and psychological stability [69,97,107].

### 7.3. Nutritional and Recovery Strategies as Epigenetic Modulators

Within the same oscillatory logic that governs TLO, nutrition and recovery are phase-setting levers for molecular adaptation. Their role is not to add stress, but to tune substrate availability and cofactor flux so that chromatin is receptive precisely when mechanical and metabolic stimuli arrive. In practice, you calibrate the amplitude, frequency, and timing of food and rest to beat in time with the AMPK–mTOR–CaMKII–SIRT1 network [99,108].

Macronutrient oscillation—alternating carbohydrate-rich sessions with low-glycogen work—shifts AMP/ATP and NAD^+^/NADH ratios, directly modulating AMPK and SIRT1. Depletion → refeeding windows also move acetyl-CoA, favoring histone acetylation (e.g., H3K27ac) at oxidative and neurotrophic loci during novelty/high-intensity phases. Strategic carbohydrate restoration then re-engages mTOR, consolidating anabolic transcription. Leucine and well-timed protein act as an anabolic “impulse”—but only in the recovery phase; delivered too early, they short-circuit energy-economy signals that support plasticity [57,98,99,108,109,110,111,112].

At finer resolution, the one-carbon and redox context shapes the same pathways. Methyl-donor supply (folate, choline, betaine) conditions SAM pools for DNA/histone methylation; NAD^+^ salvage routes (e.g., via niacin/NAMPT) influence SIRT1; polyphenols and short-chain fatty acids (e.g., butyrate) engage chromatin-modifying enzymes. None of these is a standalone fix—the timing is the intervention. Two guardrails follow: avoid chronic low-glycogen states that flatten epigenetic responsiveness, and avoid “smoothing” the oscillation with constant stimulatory nutrition that blunts the very variability the system needs [99].

Chrononutrition and sleep close the loop. Aligning meal timing and feeding windows with the circadian rhythm stabilizes clock-controlled genes that couple metabolism to stress reactivity. Sleep—especially slow-wave—provides the consolidation window for the transcriptional responses initiated in training; misaligned sleep decouples energetic state from neural function and blunts plasticity even when external load is appropriate. In practical terms, caffeine management, light exposure, and feeding windows should be treated as phase parameters, not mere habits.

Recovery can include adjunct stimuli. Heat (sauna, hot water) supports heat-shock proteins and mitochondrial signaling; intermittent hypoxia/altitude engages HIF pathways and can pre-activate oxidative programs; cooling tames acute inflammation but—crucially—can blunt mTOR peaks after hypertrophy-oriented work if mis-timed. Think of these tools as timbres that refine phase, not as solo instruments that rewrite the score.

Operationally, a simple micro-choreography is effective: (1) an intense low-glycogen session opens the AMPK/SIRT1 window; (2) carbohydrate + leucine-rich protein in the recovery phase locks in mTOR and acetylation; (3) circadian-aligned sleep consolidates; (4) a low-volume/technique day re-stabilizes coherence. Repeating this cycle keeps chromatin “elastic” and stabilizes behavior (motivation, self-regulation), directly preparing the ground for the integrated monitoring in Section 7.4 (T1–T4 windows and composite indices). In short, calibrated variability in fuel and recovery is an epigenetic intervention in its own right—the means by which epigenetic resonance can be preserved, thereby supporting durable adaptation [57,90,91,106].

### 7.4. Biomarkers and Predictive Analytics

The practical implementation of the molecular–behavioral coupling model requires biomarkers capable of tracking how training load oscillation (TLO) simultaneously engages metabolic, epigenetic, and psychological processes. Conventional markers such as lactate or cortisol reflect physiological stress but cannot capture the dynamic coupling between energy metabolism and behavioral state. The recent expansion of molecular monitoring—circulating DNA methylation, histone acetylation, and microRNA profiling—now allows this interface to be quantified, providing a bridge between metabolism and motivation [54,57,90,113].

#### 7.4.1. Core Panel

Within this framework, five adaptive domains summarize the main molecular and behavioral dimensions modulated by TLO:

1. Metabolic domain–centered on *PGC-1α*, *NR4A1*, and *SIRT1*, which translate energetic flux and redox balance into mitochondrial and vascular remodeling. Oscillatory training promotes PGC-1α demethylation, SIRT1 activation, and enhanced acetylation (H3K27ac) at oxidative gene loci, defining the molecular substrate of metabolic flexibility [54,88,113].
2. Neurotrophic domain–represented by *BDNF* and its regulatory microRNAs (*miR-132*, *miR-134*). These markers indicate the coupling between metabolic stress and neural plasticity, mediating motivation, attention, and learning. Their rhythmic upregulation mirrors the cognitive and emotional engagement associated with variable training. It should be noted that neurotrophic markers sampled in blood or saliva primarily reflect peripheral correlates of central processes. While circulating BDNF and miR-132/134 show meaningful associations with cognitive and motivational states, their relationship to hippocampal and cortical CaMKII-α/β–CREB signaling remains indirect, and cross-tissue correspondence is an open empirical question [54,55,94,95,113,114].
3. Neurotransmitter domain–comprising dopaminergic (*COMT*, *DRD2*) and serotonergic (*SLC6A4*) genes. Epigenetic shifts at these loci reflect adaptive tuning of reward sensitivity, emotional stability, and self-control—key components of performance consistency [102].
4. HPA/Stress domain–dominated by the *NR3C1* promoter, encoding the glucocorticoid receptor. Its reduced methylation under structured variability signifies improved cortisol regulation and stress resilience [107].
5. Behavioral domain–the integrative expression of all preceding layers, observable through composite indices that merge molecular resonance (e.g., *BDNF* ↓DNAm, *SIRT1* ↑) with psychometric indicators of motivation, adherence, and self-regulation [103].

**Figure 4:**
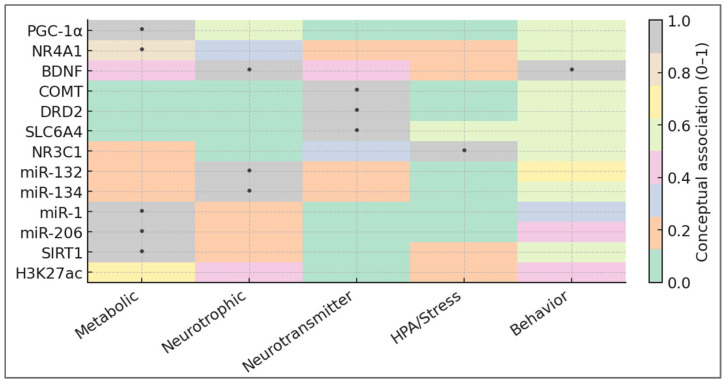
Conceptual map linking candidate biomarkers to adaptive domains. Heatmap (0–1, illustrative weights) situates PGC-1α/NR4A1/SIRT1, BDNF/miR-132/miR-134, COMT/DRD2/SLC6A4, NR3C1, and behavioral composites across the five adaptive domains—metabolic, neurotrophic, neurotransmitter, HPA/stress, and behavioral—outlining the molecular architecture of adaptive monitoring. Black dots indicate reference (anchor) biomarkers within each adaptive domain. This figure is schematic and intended for conceptual illustration rather than empirical quantification.

To operationalize the systems framework introduced above, the biomarker panel must reflect how training load oscillation (TLO) translates energetic variability into molecular and behavioral adaptation. The goal is not to catalog isolated molecules, but to map the functional domains through which metabolic and psychological oscillations communicate. These domains—metabolic, neurotrophic, neurotransmitter, HPA/stress, and behavioral—form the scaffolding of the adaptive system. These five adaptive domains and their representative molecular markers are illustrated in Figure 4, which depicts the integrative topology of metabolic, neurotrophic, neurotransmitter, stress-related, and behavioral regulation within the TLO framework.

The figure highlights the integrative nature of adaptive regulation. Each domain anchors a distinct but interconnected layer of the system: metabolic sensors set energetic tone; neurotrophic signals translate it into neural plasticity; neurotransmitter balance stabilizes motivation; and stress-axis calibration ensures behavioral coherence. Their spatial proximity in the map symbolizes resonance—adaptation emerging not from magnitude, but from alignment across molecular and psychological levels.

#### 7.4.2. Acquisition and Timing

Because epigenetic responses follow rhythmic kinetics, sampling must align with the temporal logic of TLO microcycles. Four standardized time points capture both acute and delayed adaptation phases:

- T1 (Baseline): rested morning before training load.
- T2 (Acute): 2–4 h post high-intensity or low-glycogen session, reflecting metabolic stress
- T3 (Delayed): next-morning sample showing transcriptional consolidation.
- T4 (Recovery): after a rest or low-intensity day, representing re-stabilization.

Tracking these windows across successive microcycles reveals whether metabolic and molecular phases remain synchronized. Stable oscillations in *SIRT1* activity, *BDNF* demethylation, or *miR-132* expression indicate resonance; blunted or erratic cycles signal misalignment and potential maladaptation [54,55,56,93,94,113,114]. This distinction is crucial, as peripheral biomarkers provide valuable windows into systemic adaptation but should not be interpreted as direct proxies for neural CaMKII-α/β or CREB–BDNF dynamics.

#### 7.4.3. Predictive Analytics

Integrating molecular, physiological, and psychological data transforms athlete monitoring from descriptive to anticipatory. Mixed-effects or machine-learning models can use short-term changes in molecular markers to forecast mood, adherence, or overreaching risk. A practical construct is the Resonance Index (RI)—a composite z-score summarizing adaptive molecular changes (*PGC-1α* ↓DNAm, *BDNF* ↓DNAm, *SIRT1* ↑, *H3K27ac* ↑, *miR-132* ↑) minus maladaptive ones (*NR3C1* ↑, *SLC6A4* ↑) [54,55,56,104,107,113,114].

A falling RI despite stable external load indicates loss of internal coherence and should prompt adjustments in oscillation amplitude, recovery, or cognitive load rather than simple intensity reduction. Conversely, a rising RI under variable training reflects efficient entrainment of metabolic and behavioral rhythms. In this context, the identification of circulating and tissue-specific molecular indicators can provide a quantitative interface between physiology and behavior. Table 5 summarizes key candidate epigenetic biomarkers reflecting adaptive responses to training load oscillation, highlighting their potential utility for precision monitoring and personalized conditioning.

By aligning biomarker assessment with training rhythm, these measures evolve from static indicators into real-time diagnostics of biological coherence. Rather than identifying fatigue after it appears, they reveal whether the athlete’s molecular and behavioral rhythms remain “in tune”—a dynamic signature of adaptive resonance.

### 7.5. Translational and Ethical Considerations

The systems view developed here reframes performance optimization as a problem of coherence rather than magnitude. Effective preparation depends on the phase alignment of metabolic, epigenetic, and behavioral rhythms—so that training load, cognition, nutrition, and recovery operate as coupled oscillators rather than disconnected inputs. Practically, the task is to calibrate amplitude, frequency, and timing of these oscillations so they reinforce one another [115,116,117].

In applied settings, metabolic periodization (alternating high- and low-glycogen sessions with structured recovery) should be synchronized with cognitive load (periods of focused attention, visualization, or mindfulness) and nutritional timing/composition that tune key cofactors (NAD^+^, acetyl-CoA, SAM). When these cycles converge on shared nodes—AMPK, SIRT1, BDNF, NR3C1—they amplify adaptive signaling while reducing energetic cost. Sleep and circadian alignment close the loop, stabilizing the rhythmic pattern that underpins both physiological efficiency and motivational stability [54,108,109,110,111,112,113].

The translational promise of such personalization carries ethical responsibilities. Epigenetic profiles describe dynamic states, not fixed capacities; they should guide adjustments in load variability, nutrition, and recovery—not selection or exclusion. Privacy protection, informed consent, and context-aware interpretation are mandatory, as is reporting trajectories (change over time) rather than treating single biomarkers as deterministic labels [118,119].

Finally, the same oscillatory logic extends beyond elite sport: rehabilitation, aging, and mental-health contexts also benefit from restoring rhythmic coherence between metabolism, stress systems, and cognition. In this broader sense, training load oscillation functions as a general paradigm of adaptive resilience—showing that durable stability arises not from constancy, but from variability brought into resonance. Related computational modeling, data-driven decision frameworks, and applied sports-nutrition literature provide complementary translational support for individualized adaptation, load management, and fueling strategies, without altering the conceptual scope of the present framework [120,121,122,123,124].

Taken together, these translational considerations highlight that the practical value of TLO does not lie merely in its capacity to modulate molecular pathways, but in its ability to impose structure and coherence on the temporal dynamics of adaptation. Yet a model of this scope must remain open to empirical challenge. The conceptual framework proposed here generates several predictions precise enough to be tested—predictions whose confirmation would strengthen the notion of epigenetic resonance, and whose failure would meaningfully constrain it. These critical tests are outlined in the following section.

### 7.6. Falsifiable Predictions and Critical Tests

A theoretical framework is meaningful only to the extent that it can be challenged. The model proposed here—linking TLO to molecular entrainment and behavioral coherence—generates several predictions precise enough to be tested, and therefore falsified, within controlled designs. Rather than prescribing rigid protocols, the aim is to outline the types of empirical contrasts that would decisively confirm or reject the central assumptions of the model.

Prediction 1—Phase-separation is necessary, not incidental.

If the framework is correct, biologically distinct pulses (e.g., AMPK/SIRT1-dominant, mTOR-dominant, CaMKII/CREB-linked) must retain temporal spacing for their molecular signatures to remain interpretable. When these pulses are compressed or reordered so that stress and recovery windows collide, the characteristic T1–T4 dynamics—particularly the delayed BDNF crest and SIRT1-linked consolidation—should diminish.

A falsification would occur if microcycles with intact spacing and microcycles with scrambled spacing produce indistinguishable molecular or behavioral trajectories, despite equivalent weekly work. In such a scenario, phase coherence—the backbone of the resonance concept—would lose explanatory value.

Prediction 2—Amplitude matters beyond weekly averages.

The model asserts that amplitude of oscillation (A_TLO_) is not a stylistic choice but a driver of epigenetic accessibility. Two training periods matched for total load but differing in amplitude should therefore diverge in signatures such as PGC-1α demethylation, BDNF promoter accessibility, miR-132/134 profiles, and global H3 acetylation.

This claim would be falsified if oscillatory amplitude produces no meaningful divergence in epigenetic or behavioral endpoints under equal weekly workload—implying that variability is not mechanistically privileged, and the epigenetic system responds primarily to cumulative stress.

Prediction 3—The temporal lag between CaMKII pulses and the BDNF crest is obligatory.

Central to the resonance framework is the idea that intensity-driven calcium signaling (CaMKII/CREB) and neurotrophic consolidation (BDNF) are temporally offset rather than synchronous. The BDNF rise should consistently appear in the consolidation window (T3), not during the acute spike (T2).

This prediction would be falsified if the temporal lag collapses—for example, if BDNF peaks acutely or fails to show any structured progression across T1–T4 regardless of stimulus. Such a result would challenge the notion that TLO entrains gene expression rhythms rather than simply elevating average neurotrophic tone.

Interpretive value-These tests do not aim to “prove” the model but to clarify its boundary conditions. If phase separation, amplitude, or temporal lag turn out to be dispensable, the notion of epigenetic resonance would require reformulation. Conversely, if the predicted divergences appear reliably under controlled variation in TLO parameters, the model gains empirical weight and can serve as a foundation for precision training strategies that intentionally sculpt the temporal architecture of metabolic and cognitive stimuli.

#### Integrated Summary of the TLO Framework

**Figure 5:**
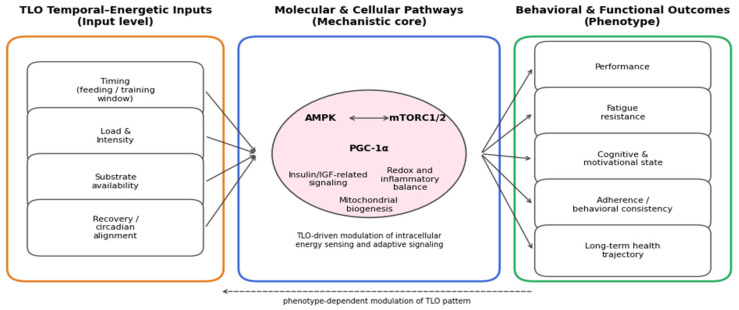
Summary of the TLO framework linking temporal–energetic inputs, molecular and cellular pathways, and behavioral–functional outcomes. The left panel (TLO temporal–energetic inputs, input level) depicts key parameters that define the oscillatory stimulus: timing of feeding and training (window), load and intensity, substrate availability, and recovery/circadian alignment. The central panel (molecular and cellular pathways, mechanistic core) illustrates the integrative signaling hub, in which AMPK, mTORC1/2, PGC-1α, mitochondrial biogenesis, insulin/IGF-related signaling, and redox–inflammatory balance form a coordinated network of energy sensing and adaptive regulation. The right panel (behavioral and functional outcomes, phenotype) summarizes the downstream expression of this network as performance, fatigue resistance, cognitive and motivational state, adherence/behavioral consistency, and long-term health trajectory. The dashed feedback arrow indicates that behavioral patterns may, in turn, modulate subsequent TLO parameter configuration, closing the loop between energetic inputs and phenotype.

At a deeper level, the framework developed here challenges the implicit separation between mechanism and behavior by treating adaptation as a distributed property of a temporally organized system. In this view, energetic oscillations do not merely trigger downstream responses, but impose constraints that shape how molecular signaling, epigenetic plasticity, and behavioral organization cohere across time. Adaptation thus emerges not from isolated pathways, but from the alignment of processes operating at different scales, each conditioning the others. Figure 5 offers a synthetic representation of this perspective, bringing together temporal–energetic parameters, molecular and cellular integration, and behavioral–functional phenotypes within a single conceptual space, and thereby clarifying how coherence—rather than mere accumulation—may serve as an organizing principle of TLO-driven adaptation.

## 8. Conclusions

Training Load Oscillation offers a conceptual framework for interpreting how structured energetic variability may coordinate molecular and behavioral adaptation via epigenetic plasticity. By prioritizing temporal organization over cumulative load, this perspective reframes adaptation as a phase-dependent process and yields testable predictions for integrated physiological and behavioral research.

## Figures and Tables

**Table 1:** Representative evidence linking exercise-induced epigenetic modifications with metabolic and neurobehavioral adaptations.

Study	Population/Model/Sample Size	Exercise/Intervention	Biological Sample	Epigenetic Marker	Gene(s)/Pathway	Direction of Change	Main Outcome/Relevance
Barrès et al., 2012 [5]	Human, trained males, *n* = 14	Acute cycling (1 h, 70% VO_2_max)	Skeletal muscle	DNA methylation	PGC-1α, PDK4, PPARδ	↓ methylation	Increased oxidative gene expression after exercise
Seaborne et al., 2018 [3]	Human, resistance training, *n* = 8	7 wk RT + detraining + retraining	Skeletal muscle	DNA methylation (450 K array)	Global, PGC-1α, MEF2A	Persistent ↓ methylation	“Muscle memory” of training via retained epigenetic marks
Denham et al., 2016 [19]	Human, young man *n* = 8	8 wk supervised resistance training	Peripheral blood	Genome-wide DNA methylation	GHRH, FGF1	↓ methylation	Reprogramming of leukocyte methylome associated with improved strength
Radom-Aizik et al., 2012 [47]	Adolescent, males, *n* = 12	Acute sprint exercise	PBMCs	microRNA	miR-1, miR-133a, miR-206	↑ expression	Regulation of myogenic and neurotrophic signaling
Sleiman et al., 2016 [10]	Mouse, *n* = 12	4 wk running	Hippocampus	Histone acetylation (H3K9ac, H3K27ac)	BDNF, Creb	↑ acetylation	Enhanced neurogenesis, learning, and motivation

Notes: DNAm—DNA methylation; miRNA—microRNA; PBMCs—peripheral blood mononuclear cells; RT—resistance training; ↑ increase; ↓ decrease.

**Table 2:** Representative magnitudes of exercise-induced epigenetic effects across tissues and paradigms.

Study (Year)	Population/Model/Sample Size	Design/Stimulus	Tissue	Epigenetic Endpoint	Gene/Pathway	Direction	Magnitude (Unit)	Window
Barrès et al., 2012 [5]	Trained males, *n* = 14	Acute cycling (1 h, ~70% VO_2_max)	Muscle	DNA methylation	*PGC-1α*, *PDK4*, *PPARδ*	↓	~2–10 p.p.	T2
Seaborne et al., 2018 [3]	Resistance-trained, *n* = 8	7 wk RT → detraining → retraining	Muscle	DNA methylation	*PGC-1α*, *MEF2A*	Persistent ↓	5–15 p.p.	Chronic
Denham et al., 2016 [19]	Human, young man *n* = 8	8 wk supervised resistance training	Peripheral blood	Genome-wide DNA methylation	GHRH, FGF1	↓	3–8 p.p.	Pre/Post
Radom-Aizik et al., 2012 [47]	Adolescent males, *n* = 12	All-out sprint exercise	PBMCs	microRNA	*miR-1*, *miR-133a*, *miR-206*	↑	0.5–1.5 log_2_FC	T2
Sleiman et al., 2016 [10]	Mouse, *n* = 10	Voluntary wheel running (4 wk)	Hippo- campus	Histone acetylation	H3K9ac, H3K27ac	↑	0.3–1.0 log_2_FC	Chronic
Lindholm et al., 2014 [8]	Humans, *n* = 23	Structured training	Muscle	DNAm + transcriptome	Oxidative pathways	Mixed	Locus-specific	Pre/Post
Geiger et al., 2024 [9]	Trained vs. untrained, *n* = 20 + 20	Cross-sectional	Muscle	DNA methylation	Exercise-responsive genes	Difference	3–10 p.p.	Baseline
Sexton et al., 2023 [54]	Trained men, *n*=20	High- vs. low-load RE (acute)	Muscle	DNAm + mRNA	Load-dependent loci	Load-specific	1–5 p.p.; 0.3–1.0 log_2_FC	T2
Podgórska et al., 2024 [55]	Elite volleyball, *n* = 18	Season	Plasma	microRNA	Adaptive panel	↑/↓	0.3–1.2 log_2_FC	In-season
Jankowski et al., 2024 [56]	Adults, *n* = 16	Exercise intervention	Muscle	DNA methylation	Targeted loci	↓	2–6 p.p.	Pre/Post
Beiter et al., 2024 [57]	Adults, *n* = 24	Acute/short/long endurance	Muscle	Transcriptome	Oxidative signaling	↑	0.5–2.0 log_2_FC	T2/T3

Notes: Values indicate typical ranges reported in the cited literature. Units correspond to percentage-point changes for DNA methylation, log_2_ fold-changes for RNA, and normalized fold-changes for histone marks. T1–T4 refer to baseline, acute, consolidation, and recovery windows.

**Table 3:** Conceptual propositions derived from the molecular–behavioral coupling model.

Conceptual Proposition	Expected Mechanistic Relationship	Implication for Research or Practice
The amplitude of training load oscillation determines the magnitude of AMPK–SIRT1 activation and subsequent epigenetic plasticity.	Larger oscillations in metabolic load enhance energetic sensing and cofactor flux (NAD^+^, acetyl-CoA), promoting histone acetylation and DNA demethylation at adaptive gene loci.	Optimize oscillatory amplitude to maintain high epigenetic responsiveness without inducing overtraining.
Sustained oscillatory training enhances demethylation and histone acetylation at BDNF, NR4A1, and COMT loci, facilitating motivation and resilience.	Cyclic activation of AMPK–CREB–BDNF and SIRT1–PGC-1α pathways modulates neurotrophic and dopaminergic gene expression.	Behavioral gains in persistence and focus emerge as molecular memory of metabolic variability.
Misaligned or excessive oscillations disrupt resonance between metabolic and behavioral domains, leading to maladaptation.	Chronic high load without recovery increases ROS and HPA axis strain, promoting hypermethylation of stress-regulatory genes (NR3C1, SLC6A4).	Periodization should balance metabolic stress and recovery to preserve resonance and psychological stability.
Mental conditioning techniques synchronize neural plasticity with metabolic rhythms, reinforcing epigenetic resonance.	Cognitive training (mindfulness, visualization) upregulates BDNF and modulates NR3C1 methylation, aligning neural and metabolic adaptation.	Integrating cognitive oscillation into TLO enhances motivation and stress resilience.
Individual resonance profiles defined by multi-omic and behavioral markers can predict training responsiveness.	Personalized variability in miRNA, DNAm, and SIRT1 activity correlates with adaptation magnitude and mental stability.	Multi-omics profiling enables precision training tuned to each athlete’s bioenergetic rhythm.

Notes: DNAm—DNA methylation; ROS—reactive oxygen species; HPA—hypothalamic–pituitary–adrenal; TLO—training load oscillation.

**Table 4:** Methodological outlook for empirical validation of the proposed molecular–behavioral coupling model.

Research Focus	Recommended Design/Approach	Key Variables and Measurements	Expected Outcome	Relevance
Epigenetic effects of training load oscillation (TLO) amplitude	Longitudinal human trial with alternating high vs. low oscillation microcycles (6–8 weeks)	AMPK–SIRT1 activation, DNA methylation (PGC-1α, NR4A1), miR-1/206 expression	Higher oscillatory amplitude → greater demethylation and miRNA variability	Defines optimal range of oscillatory stress for adaptive plasticity
Neural–epigenetic linkage via BDNF signaling	Combined exercise–neurocognitive intervention, fMRI + blood sampling	BDNF methylation, CREB activity, cognitive performance scores	Correlation between BDNF epigenetic state and executive function	Demonstrates behavioral translation of molecular adaptation
Individual “resonance profiles” and predictability of training response	Multi-omics and behavioral longitudinal study (*n* > 50 athletes)	Metabolomics, DNAm/miRNA panels, HRV, resilience and motivation scales	Distinct clustering of responders vs. non-responders by resonance index	Provides basis for precision training algorithms
Misalignment and maladaptation	Overreaching model with disrupted recovery cycles	Cortisol, NR3C1 methylation, mood state, fatigue markers	Chronic high load → loss of resonance, hypermethylation of stress-genes	Validates model prediction on maladaptive oscillation
Mental conditioning as synchronizing mechanism	Randomized trial: TLO + mindfulness vs. TLO only	BDNF, NR3C1, HRV, mood state, performance stability	Mindfulness reinforces resonance and behavioral coherence	Integrates cognitive modulation into metabolic framework
Cross-tissue coherence of epigenetic responses	Multi-tissue sampling (muscle, blood, saliva) in same subjects	Parallel methylation and histone acetylation analyses	Concordant epigenetic patterns across tissues	Demonstrates systemic nature of molecular-behavioral coupling

Notes: HRV—heart rate variability; DNAm—DNA methylation; fMRI—functional magnetic resonance imaging; BDNF—brain-derived neurotrophic factor.

**Table 5:** Candidate epigenetic biomarkers reflecting adaptive responses to training load oscillation (TLO).

Marker	Type	Tissue/Source	Primary Function or Pathway	Observed Response to Exercise or Stress	Practical Utility for Monitoring
PGC-1α promoter methylation	DNA methylation	Skeletal muscle, blood cfDNA	Master regulator of mitochondrial biogenesis	↓ methylation with endurance/low-glycogen training	Indicator of oxidative adaptation and metabolic flexibility
BDNF promoter methylation	DNA methylation	Whole blood, saliva	Neuroplasticity, motivation, mood regulation	↓ methylation and ↑ mRNA after aerobic exercise	Marker of neurobehavioral adaptation and resilience
NR4A1 expression	Transcriptional/epigenetic	Skeletal muscle	Stress-responsive nuclear receptor, metabolic–behavioral crosslink	↑ expression with oscillatory load and recovery phases	Reflects integrated metabolic and motivational activation
miR-132/miR-134	microRNA	Plasma, exosomes	Regulate BDNF and synaptic remodeling	↑ after variable-intensity exercise; linked to mood and focus	Circulating biomarkers of cognitive and motivational plasticity
miR-1/miR-206	microRNA	Muscle, serum	Myogenesis, AMPK–PGC-1α signaling	↑ with high-intensity bouts; ↓ during detraining	Indicator of anabolic–catabolic cycle efficiency
NR3C1 promoter methylation	DNA methylation	Blood, saliva	Glucocorticoid receptor; stress resilience	↓ methylation after structured variability/TLO	Reflects adaptive recalibration of HPA axis
SIRT1 activity	Enzymatic/post-translational	Muscle, PBMCs	NAD^+^-dependent deacetylase; redox-epigenetic integrator	↑ under metabolic stress; ↓ with overtraining	Global index of metabolic–epigenetic coupling
Global H3K27ac	Histone acetylation	Muscle, brain tissue (animal)	Chromatin opening, gene activation	↑ during high-intensity and novelty phases	Broad indicator of transcriptional readiness

Notes: cfDNA—cell-free DNA; PBMCs—peripheral blood mononuclear cells; HPA—hypothalamic–pituitary–adrenal axis; ↑ increase; ↓ decrease.

## Data Availability

No new data were created or analyzed in this study. Data sharing is not applicable to this article.

## Appendix A. Narrative Search Strategy and Scope

To ensure transparency while preserving the narrative character of this review, a concise description of the literature identification process is provided. Searches were conducted in PubMed, Web of Science (Core Collection), and Scopus between January 2000 and November 2025, using combinations of the following terms:

- exercise OR training OR periodization OR variability OR “training load”
- epigenetic OR “DNA methylation” OR “histone acetylation” OR microRNA
- AMPK OR mTOR OR CaMKII OR SIRT1 OR PGC-1α OR BDNF
- motivation OR resilience OR behavior OR personality.

Backward and forward citation tracking was applied to key mechanistic papers and relevant reviews. No restrictions were applied to study design, although priority was given to human studies reporting molecular endpoints relevant to metabolic and neurotrophic signaling. Animal studies were included when providing mechanistic insight not available in human data.

Inclusion criteria were:

(1) publication in English;
(2) experimental or observational work involving exercise, training load, stress–recovery perturbations, or metabolic manipulation;
(3) measurement of epigenetic endpoints (e.g., DNA methylation, histone marks, microRNAs, or transcriptional regulators);
(4) relevance to at least one of the core pathways in the proposed model (AMPK, mTOR, CaMKII, SIRT1, PGC-1α, BDNF, COMT/DRD2/SLC6A4, NR3C1).

Exclusion criteria were:

(1) case reports or isolated clinical anecdotes;
(2) pharmacological or disease-only models without an exercise component;
(3) studies lacking molecular or epigenetic measures;
(4) non–peer-reviewed material.

A simplified flow of evidence screening indicated that approximately ~1350 records were identified across databases, of which ~420 met initial abstract-level criteria. After removal of duplicates and relevance screening, ~150 full texts were examined, and roughly 70–80 studies contributed directly to the synthesis (acute, chronic, and mechanistic).

Because this review is narrative rather than systematic, no quantitative pooling or formal risk-of-bias scoring was performed. Instead, emphasis was placed on convergence across tissues, endpoints, and paradigms, as well as on consistency with the oscillatory framework developed in the main text.
